# Minimizing Delays in the Breast Cancer Pathway by Integrating Breast Specialty Care Services at the Primary Health Care Level in Zambia

**DOI:** 10.1200/GO.20.00083

**Published:** 2020-06-24

**Authors:** Mutumba Songiso, Leeya F. Pinder, Jabulani Munalula, Anna Cabanes, Sarah Rayne, Sharon Kapambwe, Aaron Shibemba, Groesbeck P. Parham

**Affiliations:** ^1^ Matero Level One Hospital, Lusaka, Zambia; ^2^ University of Zambia, Lusaka, Zambia; ^3^ University of Washington, Seattle, WA; ^4^ Medland Hospital, Lusaka, Zambia; ^5^ Susan G. Komen Foundation, Washington, DC; ^6^ Helen Joseph Hospital, Johannesburg, South Africa; ^7^ University of Witwatersrand, Johannesburg, South Africa; ^8^ Ministry of Health, Lusaka, Zambia; ^9^ University Teaching Hospital, Lusaka, Zambia; ^10^ University of North Carolina at Chapel Hill, Chapel Hill, NC

## Abstract

**PURPOSE:**

In Zambia, more than two-thirds of female patients with breast cancer present with late-stage disease, leading to high mortality rates. Most of the underlying causes are associated with delays in symptom recognition and diagnosis. By implementing breast care specialty services at the primary health care level, we hypothesized that some of the delays could be minimized.

**METHODS:**

In March 2018, we established a breast care specialty clinic for women with symptomatic disease within 1 of the 5 district hospitals in Lusaka. The clinic offers breast self-awareness education, clinical breast examination, breast ultrasound, ultrasound-guided breast biopsy, surgery, referral for chemoradiation, follow-up care, and electronic medical records.

**RESULTS:**

Between March 2018 and April 2019, of 1,790 symptomatic women who presented to the clinic, 176 (10%) had clinical and/or ultrasound indications for histologic evaluation. Biopsy specimens were obtained using ultrasound-guided core-needle procedures, all of which were performed on the same day as the initial visit. Of the 176 women who underwent biopsy, 112 (64%) had pathologic findings compatible with a primary breast cancer, and of these, 42 (37%) were early-stage (stage I/II) disease. Surgery for early-stage cancers was performed at the district hospital within 2 weeks of the time of definitive pathologic diagnosis. Patients with advanced disease were referred to the national cancer center for multimodality therapy, within a similar time frame.

**CONCLUSION:**

Breast care specialty services for symptomatic women were established in a district-level hospital in a resource-constrained setting in Africa. As a result, the following time intervals were minimized: initial presentation and performance of clinical diagnostics; receipt of a definitive pathologic diagnosis and initiation of surgery; receipt of a definitive pathologic diagnosis and referral.

## INTRODUCTION

Breast cancer is the most common malignancy in women in sub-Saharan Africa (SSA), and its incidence and mortality rates are increasing.^1,2^ Breast cancer survival rates among women in SSA are significantly lower than in western countries, primarily because of late-stage disease at the time of presentation and limited access to quality treatment.^3,4^ Late-stage presentation is multifactorial in its etiology,^5^ including low levels of community awareness, folk beliefs about the origin of the disease, lack of effective public health policies, ill-equipped health care facilities, poor access to affordable care, and workforce shortages.

### Background

Globally, breast cancer kills > 500,000 women annually.^1^ In resource-constrained environments, the vast majority of women are diagnosed at an advanced stage of disease and their 5-year survival rates range from 10% to 40%. In high-income settings, where early detection and treatment are widely available and accessible, 5-year survival rates for early-stage breast cancer exceeds 80%.^5^

Breast cancer can be detected early using 2 strategies: screening and early diagnosis. Screening involves the systematic use of testing (eg, mammography) across an asymptomatic population to detect and treat cancers. Early diagnosis, based on awareness of signs and symptoms associated with cancer, involves the recognition of possible warning signs of cancer followed by prompt action.^6,7^

How best to organize the full range of services needed for early diagnosis and treatment in low- to middle-income countries (LMICs) has not yet been determined. The benefits of centralizing all oncologic services in single urban centers (ie, comprehensive cancer centers) are well documented.^8^ The pursuit of this model in Africa, however, has resulted in approximately 80% of all cancer centers and cancer treatment facilities being concentrated in only 5 of its 51 countries: South Africa, Algeria, Morocco, Egypt, and Tunisia.^8^ In less-resourced African nations with large and widely dispersed rural populations, this organizational approach often results in long travel distances and patient queues, low patient-adherence rates, health care worker burnout, and frequent breakdown of equipment. Centralizing complex, high-technology services in a single center, and decentralization of others, may be a more effective method of distributing cancer care in resource-limited environments.^9^

Zambia is an LMIC in SSA with a population of 17.4 million people. Widespread and extreme peri-urban and rural poverty and high unemployment levels remain significant problems there. Approximately 55% of Zambia’s population lives below the international poverty line of $1.90/d, and the adult female literacy rate is 56%.^10^

### The Structure of Zambia’s Health Care System

**FIG 1:**
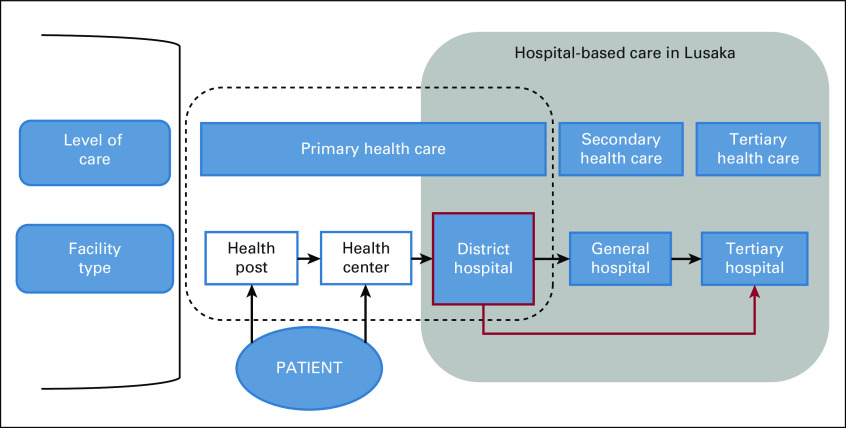
Structure of the health care and referral system in Zambia.

Zambia’s primary health care system (Fig 1) consists of rural health posts (staffed by community health assistants and nurses), health centers (staffed by clinical officers or midwives and nurses), and district hospitals (staffed by specialists: general surgeons, obstetricians and gynecologists, pediatricians, and internists). Because of human resource shortages, district hospitals in rural areas are staffed by general medical officers in lieu of specialists. District hospitals make referrals to general and tertiary hospitals, the latter of which there are 2 in Lusaka, the capital city of Zambia.

### Zambian District Hospitals

In line with WHO recommendations for improving access to health care services in LMICs, the Zambian government enacted policy in 2016 to upgrade several large health centers to district hospitals. An example is the Matero Level One Hospital, a 135-bed health facility located in the capital city of Lusaka; it serves a catchment population of 460,000. Previously a large health center, this newly upgraded health facility now serves as the referral center for 2 health centers and 5 health posts. With the addition of medical specialists, it now provides surgery, obstetrics and gynecology, internal medicine, and pediatric services (Table 1). More than 800 patients per day are evaluated in the Outpatient Department and > 50 women per day use professional obstetric services for prenatal care and deliveries; this is almost triple the numbers of clients served before the upgrade.

**TABLE 1:**
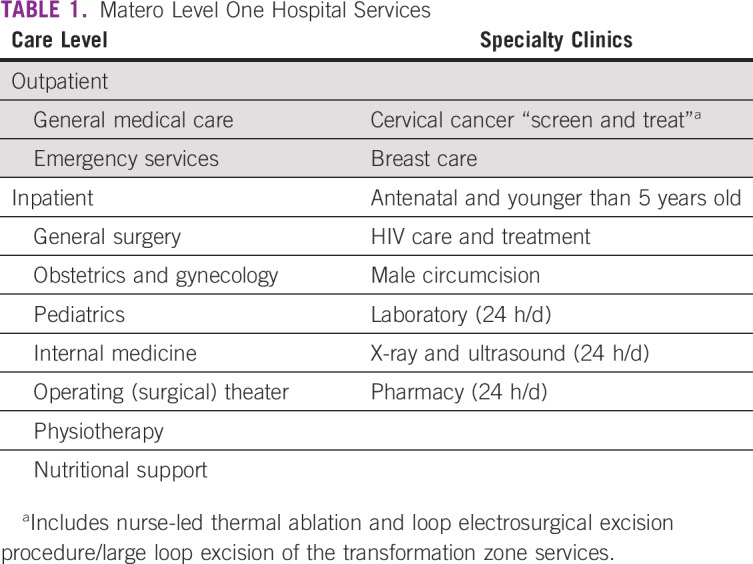
Matero Level One Hospital Services

### Breast Cancer Services

Breast cancer has the second highest cancer incidence and mortality rates among women in Zambia, after cancer of the cervix.^1^ Currently, there is 1 dedicated cancer care center, Cancer Diseases Hospital (CDH), in the country. Located in Lusaka, it serves the entire population, which is dispersed across a total area of 752,612 km^2^. Supported by 7 full-time, formally trained and board-certified Zambian clinical oncologists, the center is the country’s sole source of external beam radiation, brachytherapy, chemotherapy, and surgical oncology services. A surgical theater, added to the facility in 2018, is supported by 1 gynecologic oncologist and 1 surgical oncologist. Most patients seeking breast cancer care must move through multiple levels of the health care system before they are finally referred to CDH. The result is often prolonged delays between symptom recognition, presentation, diagnosis, and treatment, exacerbated by low levels of awareness among providers at the lower rungs of the health care system.^11^

### Preliminary Activities

Activities that have been critical to the development of breast cancer control services in Zambia include policy development, a national situational assessment, workforce training, and demonstration projects.

#### Policy development.

Zambia’s National Cancer Control Strategic Plan (2016-2021) prioritizes malignancies of the breast, cervix, prostate, and eye (retinoblastoma) and calls for a collective reduction in the cancer burden of 30% by 2030. In-country training to increase the oncology workforce is emphasized.

#### National situational assessment.

A 2014 assessment of breast health services in Zambia revealed that clinical breast examination and diagnostic (incisional and excisional biopsy) services existed at the provincial level, but use was low. Cancer treatment availability at provincial- and tertiary-level health facilities was severely curtailed by shortages of appropriately trained mid- and high-level health care personnel, inadequate pathology services, and centralization of advanced therapies (ie, chemotherapy, radiation, and oncologic surgery) at the national level.^11^

#### Training.

In 2016, efforts to expand capacity for breast cancer care in Zambia were initiated and consisted of training cervical-cancer screening nurses to provide breast cancer education and perform clinical breast examination on age-appropriate women attending cervical cancer prevention clinics; teaching diagnostic breast procedures (ie, ultrasound and ultrasound-guided core-needle breast biopsy) to general surgeons; developing a curriculum to address breast cancer management knowledge gaps and surgical skill deficiencies among general surgeons; and expansion of surgical capacity through a South-South breast surgical oncology fellowship for Zambian general surgeons at the University of Witwatersrand (the Helen Joseph Breast Care Center and the Chris Hani Baragwaneth Academic Hospital).^12^

#### Demonstration project.

Using a novel algorithm, the multistep breast cancer care pathway was successfully compressed into a single visit in 2 rural outreach camps.^13^

## METHODS

### Operationalization

Informed by the activities cited in the previous paragraphs, we established a health service platform in March 2018—the Matero Breast Care Specialty Clinic (MBCSC)—for the early diagnosis and surgical treatment of breast cancer in symptomatic women. Our primary goal was to use disease-specific workforce development and decentralization of services as strategies to expand access to breast cancer care. The clinic was operationalized by integrating its services into those routinely offered by Matero Level One Hospital (a district hospital) and equipping it with the capacity to perform clinical breast examination, breast ultrasound, ultrasound-guided core biopsy, fine-needle aspiration, breast surgery, data collection using electronic medical records, and patient referral. MBCSC operates 2 d/wk on a first-come basis (ie, symptomatic patients walk in for evaluation without prior appointments).

The demand for breast services was initially created by using the government’s “screen and treat” cervical cancer prevention program, Cervical Cancer Prevention Program in Zambia (CCPPZ), to generate referrals.^9^ Women ≥ 25 years of age attending Lusaka-based CCPPZ clinics routinely undergo clinical breast examination by cervical cancer prevention nurse providers. Those found to have abnormalities or symptoms are referred to the MBCSC for additional assessment.

**FIG 2:**
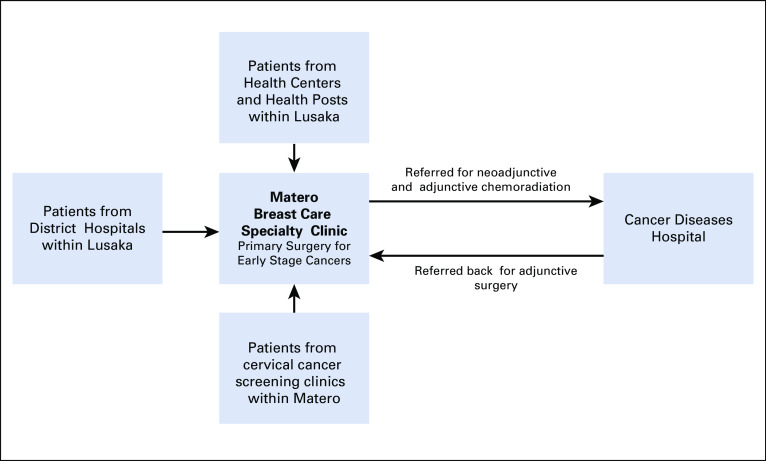
Patient flow to and from the Matero Breast Care Specialty Clinic.

As information about the clinic was disseminated, the Lusaka District Health Office enacted a new policy in December 2018 directing all primary public health facilities in Lusaka to refer women with breast symptoms or abnormal findings directly to the MBCSC (Fig 2). At the time of enrollment, all symptomatic patients, or those with abnormalities, undergo clinical breast examination by the MBCSC nurse. Those with abnormal physical findings and who are ≥ 25 years old are offered same-visit ultrasound-guided core- or fine-needle biopsy. All other cases are handled according to established standard protocols and clinical judgement of the surgeons. Biopsy specimens are transported to the University Teaching Hospital Pathology Laboratory on the same day. Specimens positive for cancer are evaluated for hormonal receptor status. Clients are recalled to the clinic when pathology reports are received. They are counseled and treatment plans determined on the basis of established protocols. Patient data are collected and stored in an electronic medical records system.

### Workforce Development

Through a South-South collaboration between the University of Zambia and University of Witwatersrand (in South Africa), two Zambian surgeons attended a 6-month, intensive, competency-based, hands-on breast management training program at Helen Joseph Breast Care Center and Chris Hani Baragwaneth Academic Hospital in Johannesburg, South Africa. A similar approach has been used to increase gynecologic oncology surgical capacity in SSA.^14^ The University of Witwatersrand breast cancer curriculum focused on 4 major areas: surgery, oncoplasty, imaging, and the multidisciplinary team approach to cancer care. One of the surgeons led the establishment of MBCSC, the subject of this report. The second surgeon only recently completed his training and is presently establishing a similar clinic in the southern part of Zambia.

To meet the increasing demand for services, a local public-private partnership was formed that provided a surgical oncologist from a local private-sector hospital to help staff MBCSC, thereby doubling the capacity for diagnostic and surgical procedures. The private-sector surgeon works at MBCSC 6 h/wk and his facility discounts surgical procedures and selected diagnostic services (eg, diagnostic mammography, histopathology, immunochemistry) for MBCSC patients as part of their corporate social responsibility.

## RESULTS

**FIG 3:**
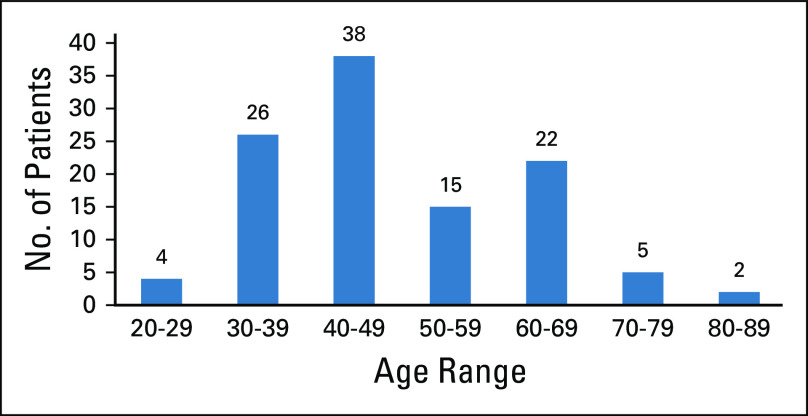
Age distribution of patients with breast malignancy (n = 112). The bar chart illustrates the age distribution of patients with histologically confirmed breast cancer. The majority (60%) are < 50 years old.

In a period of 13 months (March 2018 to April 2019), 1,790 symptomatic breast clients enrolled in the new specialty clinic, of whom 176 (10%) underwent ultrasound-guided core-needle biopsy; of those, 112 (64%) had malignant disease. Figure 3 illustrates the age distribution of clients with breast cancer, 60% of whom were < 50 years of age. Breast cancer stage at diagnosis was assessed using the American Joint Committee on Cancer TNM staging system. Clinical staging and ultrasound were the methods used to ascertain stage. As shown in Table 2, 37% of women presented with early-stage (stage I/II) disease. Histopathologic subtypes are listed in Table 3. Immunohistochemistry results were available for 50 of the 112 specimens (44.6%). The most common receptor types were estrogen or progesterone receptors (57%), followed by triple-negative (31%) type, as shown in Table 4.

**TABLE 2:**
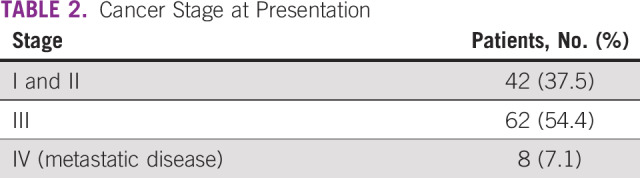
Cancer Stage at Presentation

**TABLE 3:**
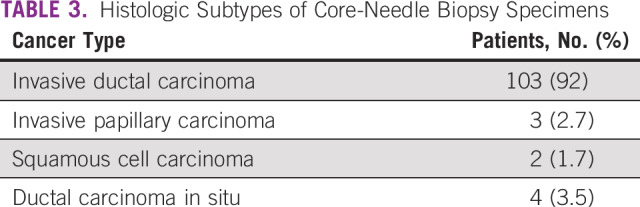
Histologic Subtypes of Core-Needle Biopsy Specimens

**TABLE 4:**
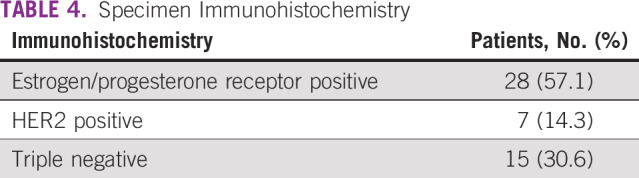
Specimen Immunohistochemistry

During the observation period, 141 surgical breast procedures were performed, of which 61 (43%) were for malignant disease and 80 (57%) for benign disease (Table 5). Of the 42 patients who presented with stage I/II disease, 95% were treated with mastectomy and 5% with conservative breast surgery (ie, lumpectomy); 40 are alive and well with no evidence of disease (range, 6 to 18 months) and 2 were lost to follow-up after surgery. Of the remaining 70 patients with locally advanced and metastatic breast cancer (stage III/IV), 18 (25.7%) completed neoadjuvant chemotherapy followed by adjunctive surgery; 28 (40%) are currently receiving chemotherapy; 6 (8.6%) are lost to follow-up; and 18 (25.7%) have died, including all patients with metastatic disease, 2 of whom died before commencement of neoadjuvant chemotherapy. All cancer surgical patients had level 2 axillary lymph node dissections, except for 5 who underwent sentinel lymph node biopsies using methylene blue dye. A total of 80 excisional biopsies for benign disease were performed, with most consisting of operations to excise giant fibroadenomas or to confirm negative ultrasound-guided core-needle biopsy findings.

**TABLE 5:**
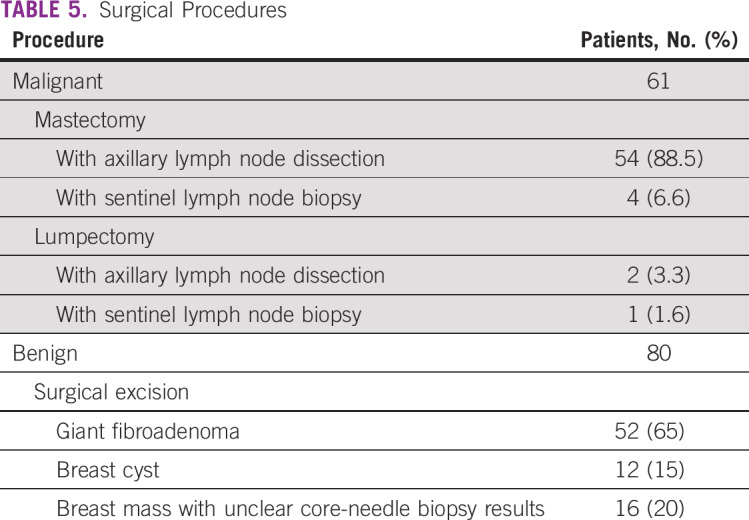
Surgical Procedures

## DISCUSSION

The WHO supports the implementation of population-based mammography screening programs in asymptomatic women, but only when capacity for confirmation of diagnoses, treatment, and follow-up of those with abnormal results exists and when resources are sufficient to meet a series of established criteria.^6,7^ This mode of early detection is not possible presently in Zambia because the facilities and resources required to undertake such a task are limited. Approximately 60% of the breast cancer cases detected in MBCSC were in women younger than 50 years. The breasts of young women are more dense and radiopaque; thus, mammography might not be as effective in detecting early disease in LMICs, where the prevalence of breast cancer in young women is 25%, compared with the rate in high-income countries, where it is 10%.^15,16^ Considering these and other factors, the next best option is early diagnosis, which increases the chances of successful treatment by discovering breast lesions in symptomatic women when the symptoms are less extensive and still curable (ie, down-staging the disease). Almost 40% of women enrolled in MBCSC presented with early-stage disease, demonstrating its potential for facilitating early diagnosis and surgical treatment of women with symptomatic breast cancer.

A unique collaboration between 2 Zambian surgeons, one based in the public sector and the other the private sector, allowed for transfer of skills and manpower resources. The partnership has been formalized with an memorandum of understanding between the public- and private-sector partnering institutions.

At the conclusion of the current study in April 2019, the MBCSC service platform was formally integrated into the Matero Level One Hospital infrastructure. Breast care specialty services are now offered routinely and consumables are provided through the government’s procurement system. This supportive action by the Ministry of Health helps ensure the sustainability of services. Equipment purchased during the study was donated to the facility by the funders.

Part of the training of the 2 Zambian surgeons in South Africa involved participation in the multidisciplinary team approach to care. This approach is being practiced at Matero Level One Hospital, although all specialties involved in patient care are not yet attending meetings.

Successes of this study include the following:

- Facilitated increased access to breast health services for symptomatic women in Lusaka
- Influenced local government policy regarding referral guidelines
- Developed a public-private partnership to build capacity in the public sector
- Minimized the time interval between presentation and diagnostic tests (ie, ultrasound and core-needle biopsy) to the same day
- Minimized the time interval between definitive pathology diagnosis and primary surgery to 2 weeks
- Minimized the time interval between definitive pathology diagnosis and referral of patients with advanced disease for chemoradiation to 2 weeks
- Incorporated a multidisciplinary approach to breast cancer care
- Early diagnosis was made for 37% of stage I/II cancers

Despite these successes, numerous challenges remain. Without confirmatory (diagnostic) mammography, it is difficult to determine the extent of disease, both in the breast with known cancer and the opposite breast. Women with early-stage disease may have the option of conservative breast surgery; however, without the information provided by diagnostic mammography, there is risk of undertreatment. In addition, because of the limited diagnostic capacity of the University Teaching Hospital Pathology Laboratory, turnaround time for histopathology results is approximately 8 weeks and routine immunohistochemistry is unavailable.

With regard to treatment, there are delays in initiating chemotherapy and radiation therapy at the national cancer center. The average time interval between patient registration and actual initiation of treatment is ≤ 12 weeks.

Horizontal integration of breast care services into other health platforms used by women deserves exploration as a means of furthering demand creation. For example, women bringing children to the district hospital for routine childhood vaccinations could be asked about breast symptoms and offered clinical breast examination by the nurses performing vaccinations, similar to the way it is conducted by cervical-cancer screening nurses. Artificial intelligence–based technology, such as computer-assisted diagnosis to triage palpable breast lumps,^17^ could help shift performance of ultrasound-guided core biopsy from high- to mid-level health care providers. The addition of onsite imprint (ie, touch) cytology of core biopsy specimens would provide women same-day provisional diagnoses for purposes of counseling and expeditious planning of future treatment.^18^ Point-of-care immunohistochemistry^19^ would represent a major contribution to treatment planning. Last, the addition of chemotherapy to the MBCSC platform would help solidify the decentralization of core therapeutic breast cancer services, with the exception of radiation therapy, bringing them closer to where women live, and further reducing some of the major system-related delays that act as barriers to early diagnosis and treatment. To ensure safety and accuracy, all these services would require stringent quality control.

In conclusion, a novel breast care service platform equipped with specialized diagnostic and surgical capabilities was made available to women with symptomatic breast disease at the primary health care level. As a result, the time interval between initial presentation and performance of clinical diagnostics (same day), receipt of a definitive pathologic diagnosis and initiation of surgery (2 weeks), and receipt of a definitive pathologic diagnosis and referral (2 weeks) were minimized. In line with government policy, efforts are underway to scale this decentralized model of breast cancer care by implementing it in other district-level hospitals in Lusaka. The workforce required to support expansion will be developed through in-country, targeted skills-building of local health professionals using core competencies–based training. Provisions will also be made for outcomes, quality assurance, and cost-effectiveness evaluations. These efforts will be undertaken by the cadre of Zambian health care professionals presently in the leadership of MBCSC.
